# PredLyP: A computational tool for predicting tissue-specific (phago-)lysosomal post-digestion peptides

**DOI:** 10.1016/j.csbj.2025.10.035

**Published:** 2025-10-22

**Authors:** Mattijn Wagt, Cristina Teodosio, Anniek L. de Jager, Jacques J.M. van Dongen, Marcel J.T. Reinders, Paula Díez, Indu Khatri

**Affiliations:** a Department of Immunology, Leiden University Medical Center, Leiden, the Netherlands; b Biomedical Research Networking Centre Consortium of Oncology (CIBERONC), Instituto de Salud Carlos III, Madrid, Spain; c Institute of Biomedical Research of Salamanca (IBSAL), Salamanca, Spain; d Department of Medicine, University of Salamanca (Universidad de Salamanca), Salamanca, Spain; e Cytometry Service, NUCLEUS, Madrid, Spain; f Translational and Clinical Research Program, Cancer Research Center (IBMCC, CSIC – University of Salamanca), Instituto de Salud Carlos III, Madrid, Spain; g Delft Bioinformatics Lab, Delft Technical University, Delft, the Netherlands; h Leiden Computational Biology Center, Leiden University Medical Center, Leiden, the Netherlands; i Department of Functional Biology (Immunology area), Faculty of Medicine and Health Sciences, University of Oviedo, Oviedo, Spain

**Keywords:** Computational tool, Post-digestion peptide, Peptide prediction, (phago)lysosomal digestion

## Abstract

Peptides are versatile tools in immunotherapy, serving as vaccines and targets for specific immunotherapeutic strategies. Peptides engage immune cells like macrophages and T cells, enabling precise modulation of immune responses. In this context, we highlight the utility of macrophages, innate immune cells involved in constant surveillance, for detecting their phagolysosomal content as a minimally-invasive biomarker strategy. Analyzing proteolytic patterns in phagolysosomes offers a high-sensitivity approach to assess tissue homeostasis and tissue disruption, such as in cancer. Despite their potential, a major challenge lies in the lack of comprehensive tools for predicting cutting sites across phagolysosomal proteases.

Therefore, we developed the computational tool PredLyP (abbreviation for “prediction of lysosomal proteases”) to identify cutting sites of phagolysosomal proteases, which are essential enzymes involved in protein degradation within (phago)lysosomes, to predict the potential peptides generated from the input proteins. Unlike existing tools, PredLyP utilizes Position Specific Scoring Matrices derived from amino acid sequences, physical (charge and hydropathy) and structural (secondary structure and solvent accessibility) features. Moreover, it incorporates a sequential cutter functionality that mimics the ordered action of proteases, providing predictive insights into substrate fragment generation. Comparisons with other tools demonstrate the superior sensitivity of PredLyP, enabling accurate prediction of complete and partial digestion fragments, a critical requirement for real-world applications in proteomics, antibody development, and immune system research. Overall, PredLyP represents a robust tool for advancing our understanding of proteolytic processes in phagolysosomes and their implications in health and disease.

## 1 Introduction

In recent years, engineered peptides and peptide-based constructs, such as synthetic vaccines, tumor-homing peptides, and peptide delivery systems, have emerged as promising therapeutic tools, particularly demonstrating significant potential in immunotherapy, such as in cancer [1], [2]. Many peptides employed in this field are derived from functional regions of proteins and possess specialized activities, such as receptor interaction, responsiveness to stimuli, cellular penetration, and modulation of signaling pathways in cells [3], [4], [5], [6], [7]. Recently, peptides have been engineered to serve as multifaceted cancer vaccines that stimulate both the innate and adaptive immune systems by engaging with immune components like neutrophils, dendritic cells (DCs), macrophages, natural killer (NK) cells, T cells, and B cells [8], [9], [10]. Additionally, peptides can act as structural units for creating advanced composite materials, incorporating features such as cell-specific targeting, responsive cleavage sequences, intracellular transport mechanisms, and therapeutic functionalities [11], [12], [13], [14], [15].

Beyond their use in therapeutic approaches, peptides are also central to the process of antigen presentation, a crucial pathway for immune surveillance. [16] This process is initiated when antigen presenting cells, such as DCs, macrophages, and B cells internalize proteins from pathogens, damaged tissue, or malignant cells. In response to inflammation in the tissues, monocytes are recruited from the bloodstream an differentiate locally into macrophages [17], [18]. In this context, macrophages engulf apoptotic cells and pathogens, forming structures known as phagosomes which then fuse with lysosomes - small cellular organelles containing proteolytic enzymes (proteases). This process results in the formation of phagolysosomes [19], cellular compartments where protein digestion occurs, generating peptide fragments [20]. Within these phagolysosomes and endosomes, generates peptides that are subsequently presented on HLA class II (HLA-II) molecules. [16] This ensures that CD4⁺ T cells can recognize and respond to foreign or danger-associated antigens. Antigen-presenting cells exploit this pathway in distinct ways: DCs excel in priming naïve CD4⁺ T cells, B cells present peptides to helper T cells to support antibody production, and macrophages integrate signals of tissue damage and infection [21], [22]. In all cases, lysosomes and phagolysosomes serve as the proteolytic compartments where specialized enzymes shape the repertoire of peptides available for HLA-II presentation. Therefore, the accuracy of phagolysomal cleavage is critical, as it shapes the final repertoire of presented peptides, making the prediction of these cleavage sites directly relevant to understanding immune responses in both cancer and infectious disease.

Several studies have indicated that a subset of these macrophages, upon completion of their functions, may recirculate back into the bloodstream via the lymphatic system [23], [24], [25]. Consequently, the application of antibody-based flow cytometry technologies could potentially enable the screening of their (phago)lysosomal contents. The identification of digested fragments derived from tissue-specific and cancer-related proteins could thus serve as a potential diagnostic and/or monitoring tool for cancer. In fact, the monitoring of circulating monocytic cells carrying tissue-specific protein fragments has been reported in patients with brain damage, including glioblastoma, brain metastasis and ischemic stroke, also allowing for prediction of glioblastoma survival [25].

To detect such tissue-specific and cancer-associated post-digestion protein fragments, using an antibody-based approach, knowledge of their amino acid sequences is essential. However, peptide sequencing technologies, such as mass spectrometry, are often expensive, labor-intensive and require high numbers of cells, not easily obtainable from patients. This challenge can be efficiently addressed through the application of computational tools. Regular expression (regex)-based tools, while useful, face limitations when dealing with complex, context-dependent patterns and can become difficult to maintain as pattern complexity increases [26], [27]. Additionally, these tools may encounter performance issues and have limited error-handling capabilities, rendering them less flexible and scalable for large or diverse datasets. Conversely, machine learning-based (ML-based) tools require extensive datasets for training and often struggle with imbalanced class distributions, potentially leading to biased or inaccurate predictions [28].

Currently, six well-established computational tools exist for identifying digestion-derived peptides or their corresponding protease-cutting sites: PeptideCutter [29], SitePrediction [30], ProCleave [31], PROSPER [32], PROSPERous [33], and iProt-Sub [34]. These tools primarily focus on enzymes commonly used in mass spectrometry analysis (e.g., trypsin, LysC) or degradative enzymes like caspases. PeptideCutter, a regex-based tool, employs regular expressions to identify cutting sites, SitePrediction uses BLOSUM-based site matching, while ML-based tools (e.g., PROSPER, PROSPERous, iProt-Sub, and ProCleave) utilize advanced machine-learning algorithms to predict the cutting sites. Despite significant computational advancements, existing tools provide limited or no support for most (phago)lysosomal proteases, largely because these enzymes often lack the large, well-curated substrate datasets required to train complex models. As a result, there remains a clear need for in silico prediction methods that can reliably model protein fragmentation by (phago)lysosomal proteases.

To address the limited protease coverage of existing tools, particularly their inability to handle (phago)lysosomal proteases with few known substrates, we developed a hybrid computational strategy that leverages the strengths of both regex- and ML-inspired approaches to predict protein fragments resulting from proteolytic cleavage by (phago)lysosomal proteases within human macrophages and DCs. Specifically, we employed a regex-based approach to predict cutting sites for proteases with fewer than 50 known substrates in the MEROPS database. For proteases with more than 50 substrates, we developed a computational method based on position-specific scoring matrices (PSSMs) [35]. Unlike conventional ML models, PSSMs can effectively model protease specificity from moderate-sized datasets without requiring extensive training or risking overfitting. This makes them particularly well-suited for underrepresented proteases, where classical ML approaches tend to fail. We integrated these components into a unified tool, PredLyP (“Prediction of cutting sites for (phago)Lysosomal Proteases”), and benchmarked it against existing public tools, demonstrating superior coverage and robust performance in macrophage-relevant protease contexts.

## 2 Material and methods

### 2.1 Identifying (phago)lysosomal proteases from multiple resources

We used the UniProt KB [36] (https://www.uniprot.org/), MEROPS [37] (https://www.ebi.ac.uk/merops/), neXtProt [38] (https://www.nextprot.org/), and Degradome [39] (http://degradome.uniovi.es/dindex.html) databases to compile a list of proteases present in human (phago)lysosomes. By applying the search criteria of “protease-specific enzymatic activity” and “lysosome subcellular location”, a total of 20 lysosomal proteases, comprising 17 endopeptidases and 3 exopeptidases, were identified (Table 1). Since exopeptidases cleave proteins and peptides at the end of a peptide chain (i.e. adjacent to a free amino or carboxyl terminus) can potentially break down proteins into monomers, only the 17 identified endoproteases were selected for designing the tool.

**Table 1:** Summary of the characteristics of twenty phagolysosomal proteases, including their type of activity (Endoprotease, Exoprotease, or both Endo/Exo), and their cleavage patterns as annotated in MEROPS, neXtProt, and the Enzyme Predictor databases. The type of activity indicates whether the protease cleaves internal peptide bonds (endoprotease) (n = 10), terminal peptide bonds (exoprotease) (n = 3), or both (n = 7). Cleavage patterns describe the specific substrate preferences or sequence motifs, as targeted by the protease.

Gene name	Protease name	Activity	MEROPS	neXtProt	Enzyme Predictor
SPPL2A	Signal peptide peptidase-like 2 A	Endo	LFT/SFLC/LF/FSLC|SLIH/FVL/LSAHG/IFGV	no information	no information
SPPL2B	Signal peptide peptidase-like 2B	Endo	LFT/SFLC/LF/FSLC|SLIH/FVL/LSAHG/IFGV	no information	no information
CTSD	Cathepsin D	Endo	x/x/x/LF|x/x/x/x	no information	x/x/x/AVLIPMFW|AVLIPMF/x/x/x
CTSV	Cathepsin V	Endo	x/x/LVI/x|x/x/x/x	Z-Phe-Arg-NHMec > Z-Leu-Arg-NHMec > Z-Val-Arg-NHMec.	no information
CTSL	Cathepsin L	Endo	x/x/LVFI/x|x/x/x/x	no information	no information
CTSK	Cathepsin K	Endo	x/x/LIVP/x|x/x/x/x	x/x/LMF/x|x/x/x/x	no information
LGMN	Legumain	Endo	x/x/x/ND|x/x/x/x	x/x/x/N|x/x/x/x	no information
CTSO	Cathepsin O	Endo	x/x/FR/R|x/x/x/x	no information	no information
CTSS	Cathepsin S	Endo	x/x/LV/x|x/x/x/x	no information	no information
CTSF	Cathepsin F	Endo	no information	x/x/FLV/x|x/x/x/x	no information
CTSZ	Cathepsin Z	Exo	no information	C-term mono and dipeptides (no action on C-term Pro)	no information
CTSA	Cathepsin A	Exo	no information	Release of a C-terminal amino acid with broad specificity.	no information
DPP7	Dipeptidyl-peptidase 2	Exo	x/x/x/PAM|x/x/x/x	Release of an N-terminal dipeptide, x/PA|x from tripeptides	no information
BACE1	Beta-secretase 1	Endo/Exo	EG/VIL/x/LF|x/AV/x/VF	E/V/N/L|D/A/E/F	no information
CTSC	Cathepsin C	Endo/Exo	x/S/x/ES|x/x/x/GR	N-term dipeptides + x/x/P2/P1|P1'/x/x/x (P2 cannot be R or K, P1 and P1' cannot be P)	no information
CTSH	Cathepsin H	Endo/Exo	x/x/x/x|x/x/x/x	x/x/x/R|x/x/x/x	no information
CTSB	Cathepsin B	Endo/Exo	x/x/x/G|x/x/G/x	x/x/R/R|x/x/x/x + C-term dipeptides	no information
CPQ	Carboxypeptidase Q	Endo/Exo	x/x/x/x|F/x/x/x	Hydrolysis of dipeptides	no information
PRCP	Lysosomal Pro-X carboxypeptidase	Endo/Exo	x/x/x/P|FA/x/x/x	x/x/x/P|x/x/x/x to release *a* C-term peptide	no information
TPP1	Tripeptidyl-peptidase 1	Endo/Exo	x/x/x/x|x/x/x/x	Release of an N-terminal tripeptide from a polypeptide, but also has endopeptidase activity.	no information

### 2.2 Predicting the cutting sites and fragments generated by (phago)lysosomal proteases

The experimentally validated substrates (full protein sequences) and corresponding cutting sites for each of the 17 endoproteases were obtained from the MEROPS database (release 12.1 as published on the 26th of April 2019). We categorized the proteases into two groups: those with more than 50 substrates available and those with fewer than 50 substrates (Table 2). For the former group, i.e. 7 endoproteases with more than 50 substrates, we developed a PSSM-based tool to predict the cutting sites of the selected proteases (Table 2). For the endoproteases with less than 50 substrates (10 endoproteases), a regex-based model was developed. Both strategies are described in more detail below.

**Table 2:** The table provides data on the 17 endoproteases, detailing the number of total substrates and non-homologous substrates listed in the MEROPS database for each protease. The final column indicates the consensus regular expression (regex) pattern associated with proteases that have a limited number of substrates. Additionally, it specifies if a Position-Specific Scoring Matrix (PSSM) was used to construct a computational model for the protease (marked in blue).

Gene name	Number of substrates	Non-homologous substrates	Consensus/PSSM
SPPL2A	5	0	LFT/SFLC/LF/FSLC|SLIH/FVL/LSAHG/IFGV
SPPL2B	5	0	LFT/SFLC/LF/FSLC|SLIH/FVL/LSAHG/IFGV
CTSD	879	379	PSSM
CTSV	1649	387	PSSM
CTSL	2888	819	PSSM
CTSK	2152	496	PSSM
LGMN	2251	701	PSSM
CTSO	2	0	x/x/FR/R|x/x/x/x
CTSS	3090	710	PSSM
CTSF	1	0	x/x/FLVK/x|x/x/x/x
BACE1	13	0	EG/VIL/x/LF|x/AV/x/VF
CTSC	31	0	x/x/x(not R or K)/ES|x(not P)/x/x/x
CTSH	33	0	x/x/x/R|x/x/x/x
CTSB	569	311	PSSM
CPQ	1	0	x/x/x/x|F/x/x/x
PRCP	3	0	x/x/x/P|x/x/x/x
TPP1	30	0	x/x/GP/FMG|F/RL/x/P

#### 2.2.1 Developing a predictor for proteases with more than 50 substrates

**Fig. 1:**
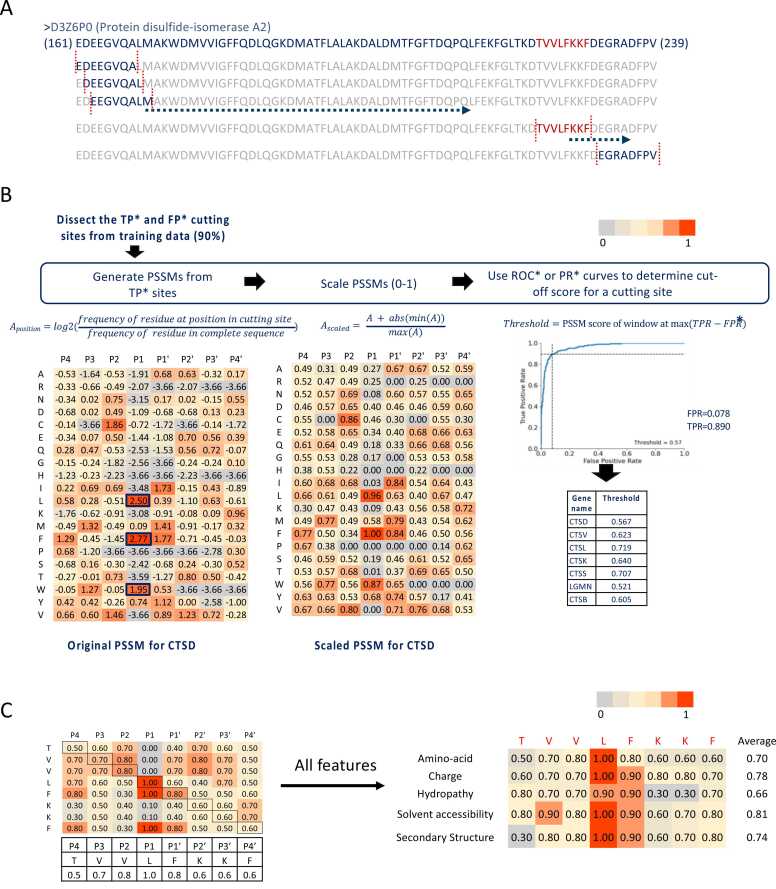
**Overview of the methodology for profiling cutting sites using the PredLyP tool.** A) The sliding window is shown with red dashed lines and a positive cutting site (sequence: TVVLFKKF) in the protein disulfide-isomerase A2 (UniProt ID: D3Z6P0) is shown in red. **B)** Position-specific scoring matrix (PSSM) representation of the cutting site. Each residue from positions P4 to P4′ is scored based on amino acid substitution probabilities learned from training data, with scores scaled between 0 and 1. This matrix highlights residue preferences at each position surrounding the cleavage site. C) All five features contributing to the prediction model (amino acid PSSM, charge, hydropathy, solvent accessibility, and secondary structure) are displayed for the cutting site. Each feature is scored across the 8-residue sliding window, and the final prediction score is computed as the average of these feature scores, as shown in the "Average" column.

For proteases with more than 50 substrates, full-length substrate proteins and annotated cleavage sites were retrieved from the MEROPS database. Each cleavage site was represented as an 8-residue window (P4–P4′) centered on the scissile bond (Fig. 1A). These windows formed the training instances from which feature-specific position frequency matrices were constructed (Fig. 1B), capturing amino acid identity, charge, hydropathy, secondary structure, and solvent accessibility at each position. After normalization, these matrices were transformed into position-specific scoring matrices (PSSMs), which served as the basis for predicting new cleavage sites (Fig. 1C).

To reduce redundancy and prevent overfitting, experimental substrates with sequence identity exceeding 70 % (standard homology threshold) were filtered out using CD-HIT [40]. The remaining non-homologous substrates were divided into training and test sets using a 90/10 split at the substrate level, ensuring that homologous proteins were not shared across sets. Models were trained on the 90 % training set using the constructed PSSMs, and predictions were then made on the 10 % held-out test set, which served as an independent evaluation dataset.

Performance was evaluated on the independent test set using classification thresholds derived from Precision–Recall curve optimization applied to the training data. Metrics included precision (TP/(TP+FP)), sensitivity (TP/(TP+FN)), specificity (TN/(TN+FP)), and F1 score (harmonic mean of precision and sensitivity). To assess the impact of class imbalance, two versions of the data were employed: the unbalanced MEROPS dataset (Set-1, reflecting the true distribution of cleavage vs. non-cleavage sites) and an under-sampled dataset (Set-2, 3:1 negative:positive ratio). Together, these evaluations allowed us to benchmark performance both under realistic conditions and under balanced settings commonly used in computational tool development.

##### 2.2.1.1 Sequence and structural features used to generate the model

We utilized various sequence (amino acid sequence, hydropathy, charge) and structural (secondary structure and solvent accessibility) features of the eight amino acid residues around the cutting site (P4-P3-P2-P1-(*cleavage site)*-P1’-P2’-P3’-P4’) and presented them each into a PSSM. Hydropathy characteristics (‘ζ’, ‘M’ and ‘Φ’ characters, representing hydrophilicity, neutral and hydrophobicity, respectively) and charge properties (‘+’ for positive, ‘N’ for neutral and ‘-’ for negative charge) were calculated from Thermo Fisher’s “Amino acid and Physical Properties” resource (accessed on 1 November 2021). Secondary structure (represented as C = chain, E = beta-strand, H = helix) and solvent accessibility (score ranging from 0 to 9, with 0 indicating not accessible and 9 indicating very accessible) were determined using SABLE v4 [41], [42], [43], [44].

##### 2.2.1.2 Generation of PSSMs for all the features

***Step I:*** Calculate the frequency of occurrence for each encoded character across the complete sequence of the substrates:

$$F=\;\sum_{c}^{\left|C\right|}\frac{\left|c\in S\right|}{\left|S\right|}$$

where:

S: The list of substrates

C: The set of all unique characters (for example, amino acid residues in the case of protein substrates).

c: An element of the set C, representing each character.

∣c∈S∣: The count of occurrences of character c in the list of substrates S.

∣S∣: The total number of substrates in the dataset.

This produces a frequency vector of 20 (amino acids), representing how often each amino acid occurs across all substrates.

***Step II:*** Calculate the frequency of occurrence for each encoded character at specific locations around the cutting site across substrates:

$$\mathrm{PFM}=\;\sum_{c}^{\left|C\right|}\sum_{p}^{\left|L\right|}\frac{\left|c\in \;{\mathrm{CS}}_{*,\;p}\right|}{\left|{\mathrm{CS}}_{*,p}\right|}$$

where:

C: The set of all unique characters (e.g., amino acids for protein substrates).

c: An element from the set C, representing each character (e.g., a specific amino acid).

L: The length of the cutting site fragment. This corresponds to the size of the window around the cleavage site (in this case, 8 amino acid residues).

p: An index representing a specific position within the cutting site window. The range of p is from 0 to L, and it represents each individual position within the window where the cleavage site occurs.

CS(∗,p): This represents a list of all substrates' cutting sites at a specific position pp within the 8 amino acid window. It is a list of the amino acids present at position pp across all substrates at their respective cleavage sites.

∣CS(∗,p): This represents the total number of substrates considered for position pp (i.e., the total number of substrates that have a cleavage site in the dataset).

When you condition on c,p, as in PFM_A,3_, this would represent the frequency of amino acid A occurring at position 3 in the cutting site window across all substrates. The result is a 20 × 8 matrix, where rows correspond to amino acids and columns correspond to positions P4–P4′ relative to the cleavage site.

***Step III:*** Calculate the frequency of a character at a given position in PFM divided by the total frequency F of that character in substrates:

$$\mathrm{PSSM}=\sum_{c}^{\left|C\right|}\sum_{p}^{\left|L\right|}\left\{\begin{array}{cc} {\;\log}_{2}\left(\frac{{\mathrm{PFM}}_{c,p}}{F_{c}}\right) & \mathrm{if}{\mathrm{PFM}}_{c,\;p}\;>0\;\wedge \mathrm{if}F_{c}>0\\ \;\mathrm{NA} & \mathrm{otherwise} \end{array}\right)\;$$

where:

C: Set of all possible characters (e.g., amino acids).

c: An element from the set C, representing a specific character (amino acid).

L: Length of the cutting site fragment (for example, an 8-residue window around the cleavage site).

**p**: The index of a specific position within the window (from 0 to L).

PFM_c,p_: Position Frequency Matrix (PFM), which is the frequency of amino acid c at position pp in the cutting site window.

F_c_: The frequency of amino acid cc in the complete substrate list.

Any NA and infinite values resulting from the log_2_ transformation are set to the minimum value of the entire matrix. Each entry represents the enrichment or depletion of an amino acid at a given position compared to its overall background frequency.

***Step IV:*** Scale the log-transformed matrix to a range of 0–1:

$${\mathrm{PSSM}}_{\mathrm{scaled}}\;=\;\frac{\mathrm{PSSM}+\mathrm{abs}\left(\min\left(\mathrm{PSSM}\right)\;\right)}{\max\left(\mathrm{PSSM}\right)\;}$$

The matrix is shifted to remove negative values and scaled between 0 and 1 to allow comparability across features such as amino acid identity, charge, and hydropathy.

***Step V:*** Use sliding window to fetch scores for each site:

To predict the likelihood of a cutting site, a sliding window of eight amino acid residues is moved across the sequence. For each position within this window, a score is calculated based on all the features: i.e., for each candidate cleavage site, an 8-element vector of scores based on the PSSM is generated, corresponding to the residues from P4–P4′, and averaged to yield a single site score between 0 and 1. An example can be seen in Fig. 1**A**. Similarly, the scores are calculated for each feature (Fig. 1**B)**; a score of 0 represents a low probability of a cutting site, while a score of 1 indicates a high probability. The cutting sites with scores above thresholds are considered to be positive.

**Step VI:** Weighing the scores of windows (sites in question) by feature importance:

Averaging the scores treats all positions equally, diminishing the impact of preferred positions. To address this, weights can be applied to the scores obtained from the windows. We utilized a Random Forest to determine these weights and feature importance; i.e., we used the Random Forest feature importances, normalized to sum to 1, as weights to rescale each feature’s contribution to the final site score:

$$\mathrm{Final score}=\;\sum_{i}^{\left|\mathrm{scores}\right|}{\mathrm{scores}}_{i}\;\times {\mathrm{feature importances}}_{i}$$

***Step VII:*** Use Precision Recall curve to calculate thresholds for predicting cutting sites:

The Precision-Recall (PR) curve was used to evaluate the performance of our model. In this context, the PR curve was used to determine the optimal threshold that separates predicted positive cutting sites (those that are likely to be cleaved) from negative sites (those that are unlikely to be cleaved), see also Fig. 1B for an explanation.

For each threshold t, precision and recall are calculated, and the optimal Precision-Recall threshold, T, is determined as the point where the trade-off between precision and recall is maximized. It provides the best trade-off between correctly identifying positive cutting sites and minimizing false positives:

$$T=\arg\underset{t}{\max}\left({\mathrm{Precision}}_{t}-{\mathrm{Recall}}_{t}\right)$$

#### 2.2.2 Developing a regex pattern for proteases with less than 50 substrates

For the second group, i.e. 10 (phago)lysosomal proteases with less than 50 substrates in the MEROPS database, we deduced a consensus cleavage site by integrating information from MEROPS and neXtProt databases (Table 1, Table 2). Regex is used to identify consensus cleavage sites by matching specific patterns of residues around the scissile bond. Cleavage site motifs often follow a structured format where residues before and after the cleavage site are denoted as P4, P3, P2, P1 (before cleavage) and P1', P2', P3' and P4' (after cleavage). Using data from MEROPS and neXtProt, these patterns are encoded into regex. For example, for BACE1, the cutting site in MEROPS is "EG/VIL/x/LF|x/AV/x/VF" and NextProt is “E/V/N/L|D/A/E/F” (Table 1), where both sources indicate variability at P4 and P1, with conserved hydrophobic residues at P1′. These patterns were aligned, and the most representative residues were retained, resulting in the consensus cleavage site "EG/VIL/x/LF|x/AV/x/VF" for BACE1 (Table 2). As another example, for CTSC, MEROPS describes the motif “x/S/x/ES|x/x/x/GR”, while neXtProt indicates restrictions that P2 cannot be R or K, and P1/P1′ cannot be P. We integrated this information into the regex string x/x/x[^RK]/ES= [^P]/x/x/x, which encodes both positive preferences (E at P1, S at P1′) and exclusions (P2 ≠ {R,K}, P1 and P1′ ≠ {P}).

#### 2.2.3 Fetching fragments from the cutting sites

**Fig. 2:**
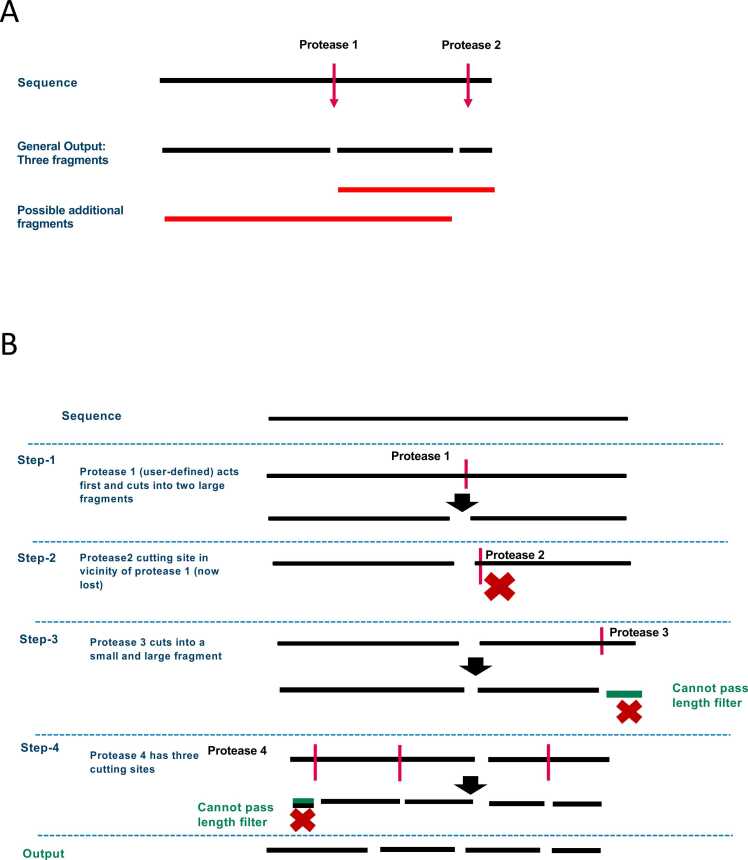
**Generating fragments from the cutting sites of the proteases.** A) Illustration of all possible fragments generated from predicted cutting sites. The black bars represent the original sequence and the completely digested fragments resulting from the protease cuts. Red bars depict additional fragments generated to account for partial digestion, allowing for combinations of adjacent segments (e.g., 1 +2, 2 +3). These partial fragments provide a more comprehensive representation of potential digestion outcomes. B) Overview of the sequential cutter process, where proteases act sequentially on the output of the previous step. In Step 1, the user-defined Protease 1 makes the initial cut, splitting the sequence into two large fragments. In Step 2, Protease 2 attempts to cut near the site of Protease 1’s action, but this site is lost due to the previous cleavage, indicated by the red "X" symbol. In Step 3, Protease 3 cleaves one of the remaining fragments into a smaller and a larger piece. In Step 4, Protease 4 acts on its assigned sites, but some resulting fragments fail to pass the minimum length filter, indicated by the red "X" (cross) symbols. The final output includes all fragments that meet the length criteria after sequential cleavage by all proteases. This approach models the stepwise digestion process and captures both complete and partial digestion scenarios.

Currently available tools focus primarily on predicting cleavage sites, but understanding the resulting peptide fragments is crucial for gaining insights into proteolytic processing. To bridge this gap, we enhanced our tool with two key functionalities. First, it can generate all possible peptide fragments that could arise from partial digestion, using the predicted cleavage sites as a basis (Fig. 2**A**). Second, it includes a sequential cutter system, which simulates proteolytic digestion as a stepwise process where proteases act in sequence rather than simultaneously (Fig. 2**B**).

This sequential approach is particularly useful in scenarios where the lysosomal concentrations of proteases vary significantly. In such cases, the most abundant protease(s) typically dominate(s) the initial cleavage events, potentially altering subsequent cleavage sites for less abundant proteases due to competition. By incorporating these functionalities, the tool offers a more comprehensive understanding of both cleavage patterns and the resulting peptide fragments under diverse proteolytic conditions.

### 2.3 Comparison with off-the-shelf ML algorithms

We compared PredLyP with several widely used ML methods. These included *Naïve Bayes* [45] i.e. GaussianNB, a probabilistic classifier with the assumption of feature independence; *Multi-layer perceptron* [46] i.e. MLPClassifier, a feedforward neural network; *Nearest Neighbors* [47] i.e. KNeighborsClassifier, a consensus classifier that identifies the K closest neighbours to the input based on the training dataset; *Decision Tree* [48] i.e. DecisionTreeClassifier, a classifier that uses a cascading set of learned if-then-else rules; and *Random Forest* [49] i.e. RandomForestClassifier, an ensemble classifier that creates multiple decision trees using randomly generated starting parameters. These ML algorithms were implemented on the one-hot-encoded Set-2.

### 2.4 Comparison to publicly available tools

Evaluation of currently existing tools revealed that information on protease-cutting sites was only available for 5/17 of the endoproteases (CTSB, CTSD, CTSL, CTSK, CTSS) (Supplementary Table S1). We compared PredLyP to the following available tools: PROSPER, PROSPERous, and ProCleave. The PROSPER comparison was conducted using the standalone version of PROSPER, while PROSPERous and ProCleave were tested using their web servers (accessed on January 20, 2022). As the other tools do not support all (phago)lysosomal proteases, the comparison was limited to a subset of the proteases supported by PredLyP. Additionally, to showcase the performance of our tool, we included the cutting sites for caspases 1, 3, 6, 7, and 8 as internal controls, since they are commonly supported and available in the majority of publicly available tools.

### 2.5 Implementation details

PredLyP was written in version-controlled software with Python 3.8. To provide public and easy access to the tool, we developed a web server with a FastAPI backend and a Celery event queue for asynchronous predictions (http://predlyp.usal.es/). The frontend was created using Angular (version 14). Here, users can submit or upload protein sequences in FASTA format. Upon submission, users receive a 201 HTTP status code, indicating successful submission. The task is then queued for processing when the server has an idle processor core. The website displays a list of recently submitted tasks and their status, allowing users to monitor progress of their own tasks and view results once completed. The results page shows the submitted sequences along with identified fragments and cutting sites (Supplementary Figure S1). For each fragment, the sequence, assigned score, molecular weight, and isoelectric point are provided. Cutting sites are listed per protease, detailing the P1 position, score, P4-P4’ window, and confidence for each site.

## 3 Results

### 3.1 Selection of substrates for training the PredLyP tool

The substrate sequences for 17 (phago)lysosomal proteases were obtained from the MEROPS database (Table 1). Among these enzymes, 7 (phago)lysosomal proteases (CTSB, CTSD, CTSK, CTSL, CTSS, CTSL, and LGMN) were identified as having more than 50 experimentally validated substrates. Following the removal of homologous sequences, we retained 543 ± 198 non-homologous substrates for each of these proteases, ranging from 311 to 819 substrates (Table 2). These non-homologous substrates were utilized for the training of the prediction tool.

### 3.2 PSSM profiles highlight positional preferences for protease-cutting sites

**Fig. 3:**
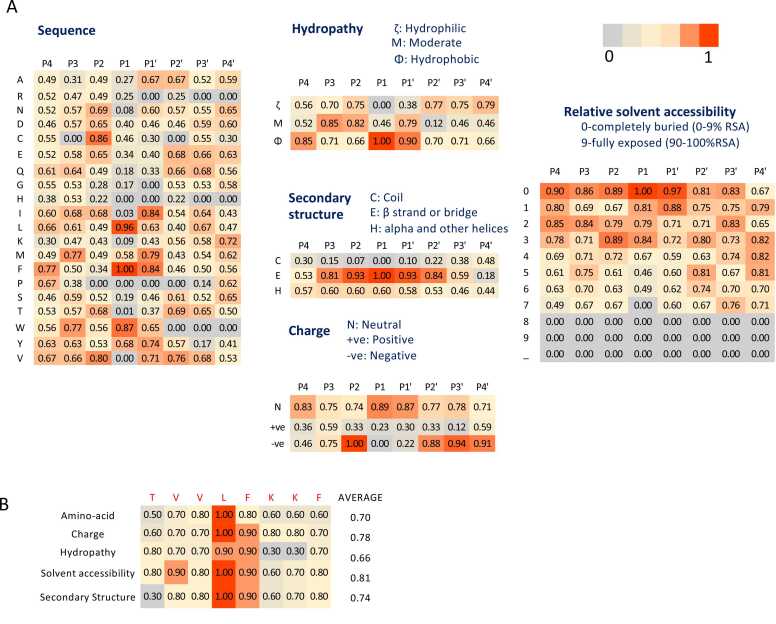
**PSSM Matrices for Five Features and Positive Cutting Site Example for Cathepsin D (CTSD)**. **A)** Representative examples of Position-Specific Scoring Matrices (PSSMs) generated for the five features of the Cathepsin D (CTSD) protease. The X-axis indicates the positions surrounding the cutting site (P4 to P4′), where the cutting occurs between P1 and P1′. The Y-axis represents the scores for each feature as detailed in the Methods section. These PSSMs illustrate the contribution of residues at each position to the protease's substrate specificity. **B)** A positive cutting site example is shown, highlighting the application of combined feature thresholds derived from the PSSMs.

We generated PSSMs for various features, including amino acid sequence, charge, hydropathy, relative solvent accessibility, and secondary structure, across all seven (phago)lysosomal proteases with more than 50 substrates. Fig. 3**A** illustrates the PSSMs for CTSD as an example. Our analysis revealed that at substrate position P1, the amino acids phenylalanine (F), leucine (L), and tryptophan (W) were strong indicators of a cleavage site for CTSD, along with cysteine (C) at position P2 and isoleucine (I) and F at position P1’. Notably in CTSD profiler, a negative charge was highly favored at position P2 compared to other positions. Hydrophobicity was preferred at P1, while beta strands in the secondary structure were strong indicators for positions P3 to P2' (Fig. 3**B**). Additionally, low solvent accessibility was generally favored over high solvent accessibility across all positions. Finally, these features were integrated to generate a comprehensive score for each site, identifying positive cutting sites based on established threshold cutoffs (determined using PR curve optimization as described in Methods, Section 2.2.1.2) (Fig. 1).

Similarly, hydrophobicity was preferred at the P2 position of the cleavage site for CTSV, CTSL, CTSS, and CTSK (Supplementary Figure S2), whereas CTSB cleavage sites predominantly featured all hydrophobic amino acids. In contrast, LGMN cleavage site displayed a preference for the amino acid asparagine (N) and hydrophilicity at the P1 position. These findings highlight the diverse, yet position-specific preferences of (phago)lysosomal proteases, underscoring their specialized roles in substrate recognition and cleavage.

### 3.3 Evaluating PredLyP performance in real-world and balanced dataset scenarios

The substrates exhibited a 70 % higher prevalence of negative sites compared to positive sites, highlighting a significant imbalance in the dataset where non-cutting sites far outnumbered cutting sites. To address this, we designated this original dataset as Set-1 and created a second dataset, Set-2, by under-sampling the negative sites to achieve a 3:1 ratio of negative to positive sites. Although we evaluated the performance of the PredLyP tool using both datasets, Set-2 does not accurately reflect real-world scenarios and was included primarily for comparative purposes, as many existing tools are developed using such balanced datasets.

Under-sampling in Set-2 resulted in notable enhancements in the F1 score, with improvements ranging from 36 % to 65 %, compared to only a 1 % improvement in Set-1 (Supplementary Figure S3A). Precision also increased substantially, increasing by 35–70 % in Set-2, compared to 1 % in Set-1 (Supplementary Figure S3B). These improvements in F1 score and precision are attributable to reduced false positives. Conversely, sensitivity and specificity (Supplementary Figures S3C and S3D), which remain unaffected by class imbalance, showed minimal variation across datasets.

Despite the observed gains in F1 score and precision in Set-2, these metrics may not translate effectively to real-world scenarios due to the dataset's artificial balance, reinforcing the importance of using Set-1 for realistic performance evaluation.

### 3.4 Evaluation of Feature Contributions to Protease Prediction Performance in PredLyP

A comparative analysis was conducted to evaluate the influence of various input feature sets on the predictive performance of PredLyP using dataset Set-1. Three scenarios were assessed: 1) using solely amino acids information, 2) combining amino acids with physical features (hydropathy and charge), and 3) integrating amino acids, physical features and structural features (secondary structure and solvent accessibility).

The results revealed that for specific proteases, such as CTSK, CTSL, CTSS, and CTSV, improvements were exhibited with the inclusion of physical features. For instance, CTSK showed a 0.6 % increase in F1 score (Supplementary Figure S4A), a 0.3 % improvement in precision (Supplementary Figure S4B), and an 8.1 % gain in specificity (Supplementary Figure S4D). Similarly, CTSL exhibited a 1.3 % increase in specificity, while CTSS and CTSV demonstrated sensitivity improvements of 3.0 % and 11.8 %, respectively (Supplementary Figure S4C). On the other hand, adding structural features provided further marginal improvements for some proteases but generally showed diminishing returns. For example, CTSB and CTSV demonstrated a slight increase in specificity, while LGMN showed small gains in sensitivity and specificity.

These findings suggest that while the integration of physical features can enhance predictions for specific proteases, the overall benefit varies across proteases, and the addition of structural features does not universally improve predictive accuracy.

### 3.5 Performance assessment of weighted features in enhancing protease prediction

We performed an analysis to determine whether feature weighing could enhance the predictive capability of PredLyP. This approach involves assigning greater importance to features crucial for predicting cutting sites while reducing the influence of less significant features. To assess the significance of each feature in predicting cutting sites, we employed a Random Forest classifier (**Methods**, Supplementary Figure 5B). This analysis determined the contribution of each feature at various positions within the P4-P4’ window.

The feature importance analysis highlighted the substantial significance of amino acid features at individual positions within the window, particularly at positions P2 and P1, which exhibited the highest importance in predicting cutting sites (Supplementary Figure S5A). In contrast, other features such as hydropathy demonstrated comparatively lower importance, albeit with notable contributions at their respective P2 and P1 positions. These findings underscore that amino acid composition plays a primary role in predicting cutting sites, surpassing the impact of additional structural and chemical features.

**Fig. 4:**
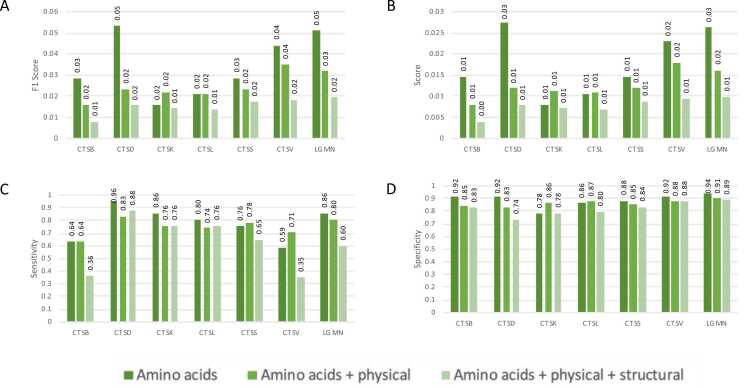
**F1 score, precision, sensitivity, and specificity comparisons on PredLyP using different input features**. Analyses were performed on the original unbalanced Set-1 dataset. Models were evaluated using 1) amino acid features alone, 2) amino acid plus physical features (charge and hydropathy), and 3) the full set of amino acid, physical, and structural features (secondary structure and solvent accessibility). A weighted version incorporating Random Forest–derived feature importances is also shown.

Upon implementing feature weighing during the calculation of the metrics, we observed average improvements of 1 %, 0.5 %, 16 %, and 3 % for F_1_ score, precision, sensitivity, and specificity, respectively, compared to the non-weighted version (Fig. 4). Despite 16 % gains in sensitivity, the weighted predictor did not outperform in other metrics for the unweighted model based solely on amino acid features. These findings suggest that while feature weighting provides minor performance enhancements, it does not justify replacing the amino acid-only approach, which remains highly effective and computationally efficient.

### 3.6 PredLyP outperforms off-the-shelf machine learning models in predicting protease-cutting sites

We compared the performance of PredLyP with several standard ML algorithms using the amino acid sequence feature set to evaluate its predictive power (Supplementary Figure S6). The GaussianNB, MLPClassifier, KNeighborsClassifier, DecisionTreeClassifier, and RandomForestClassifier classifiers (Methods) had an F1 score of 49 %, 53 %, 57 %, 52 %, and 49 %, respectively. PredLyP achieved the highest F1 score of 76 %, which is a 34.5–57.4 % improvement over the F1 scores of other methods. In terms of sensitivity, PredLyP achieved a value of 85 %, significantly outperforming the other methods, which ranged from 38 % to 55 %. While specificity and precision were also competitive for some ML algorithms, PredLyP maintained a balanced and superior overall performance, ensuring not only high sensitivity but also strong precision and specificity. This better sensitivity performance of PredLyP underscores its value in applications where capturing true cutting sites is critical.

### 3.7 Comparing PredLyP with publicly available tools for phagolysosomal protease cutting site prediction

**Fig. 5:**
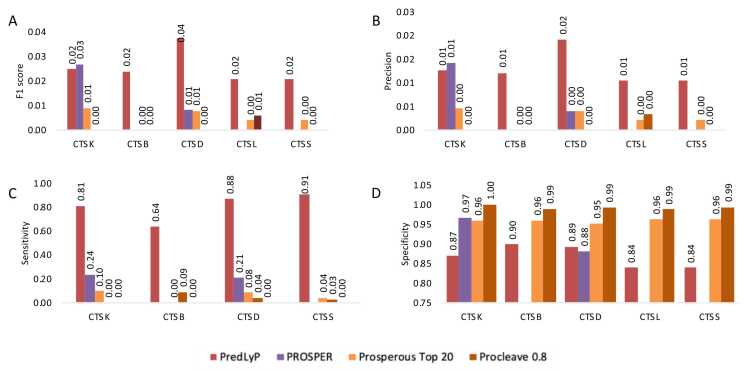
**F1 score, precision, sensitivity, and specificity comparisons between predLyP, PROSPER, PROSPERous and ProCleave using CTSB, CTSD, CTSK, CTSL, and CTSS**. Analyses were performed on the original unbalanced Set-1 dataset. PredLyP thresholds were determined using Precision–Recall optimization (see Methods), PROSPERous was evaluated at top-20 predicted sites, and ProCleave was tested at thresholds 0.8 as recommended by the tool.

We compared PredLyP with existing tools, including PROSPER, PROSPERous, and ProCleave, for predicting cutting sites in phagolysosomal proteases using real-world original unbalanced dataset (Set-1) (Fig. 5). PROSPER supports only CTSK and CTSD among the phagolysosomal proteases. When comparing PredLyP with PROSPER for CTSK predictions, PROSPER outperformed PredLyP in terms of F1 score, precision, and specificity, while PredLyP demonstrated superior sensitivity. Conversely, for CTSD predictions, PredLyP outperformed PROSPER across all metrics. For the PROSPERous and ProCleave tools, PredLyP demonstrated better performance across all evaluated proteases in terms of F1 score, precision, and sensitivity. Although PredLyP achieved specificity values above 80 % overall, its specificity was approximately 9 % lower compared to the other tools.

To further validate the reliability of our tool, we compared PredLyP with PROSPER and ProCleave for predicting cutting sites in caspases 1, 3, 6, 7, and 8. Caspases are proteases with a substantial number of substrates, as supported by MEROPS, and are widely studied using various prediction tools. PredLyP significantly outperformed both PROSPER and ProCleave in terms of F1 score, precision, and sensitivity, while maintaining comparable specificity (Supplementary Figure S7). Notably, ProCleave achieved perfect precision for caspase 6 in this dataset, though this result was based on a single cutting site prediction, making it an outlier. Where PROSPER outperformed PredLyP (e.g., CTSK), the difference likely reflects disparities in training-set composition and more conservative thresholding in PROSPER rather than overfitting in PredLyP; our sensitivity/specificity remained stable across homology filtering and class-balance settings. Additionally, it is important to highlight that while the tools generally exhibit very high specificity, their sensitivity remains limited.

### 3.8 Validation of PredLyP v2 using in-house generated mass spectrometry data on colorectal cancer

To validate the performance of PredLyP in a real-world dataset and to assess the functionality of the sequential cutter, we employed an in-house colorectal cancer mass spectrometry dataset. This dataset was generated from purified normal epithelial and tumor cell populations from seven colorectal cancer samples and comprised a total of 37,154 peptide fragments derived from 4722 substrate proteins.

The experimental workflow involved enzymatic digestion of the samples using Lys-C and Trypsin. To enable integration into PredLyP, cutting site information for these proteases was added to the tool in accordance with the established workflow used for phagolysosomal proteases. First, substrate sequences for Lys-C and Trypsin were retrieved from MEROPS and converted into PSSMs. Optimal thresholds for cleavage site prediction were then calculated as described in the Methods section.

The identified peptide fragments were mapped back to their corresponding protein substrates to extract P1 cleavage positions. These P1 locations served as the reference to evaluate whether PredLyP accurately identified experimentally observed cleavage sites. Using the sequential cutter functionality, PredLyP reproduced cleavage events across the dataset and yielded the following performance metrics: an F1 score of 0.197, precision of 0.109, sensitivity of 0.993, and specificity of 0.893.

These results demonstrate that PredLyP is capable of handling large, experimentally derived peptide datasets and that the sequential cutter accurately models sequential protease digestion events. The high sensitivity highlights the ability of PredLyP to capture true cleavage events, while the specificity reflects its robustness in minimizing false predictions. Although the relatively lower precision and F1 score reflect the highly unbalanced nature of the dataset (where negative sites greatly outnumber positive sites), this validation provides strong experimental support for the predictive capability of PredLyP.

## 4 Discussion

We developed PredLyP, an advanced predictor that integrates PSSMs to accurately identify cutting sites of phagolysosomal proteases and caspases. By utilizing PSSMs, PredLyP assigns scores to amino acid sequences based on their conservation patterns across substrates, reflecting the evolutionary constraints at specific positions within protease cleavage sites. This methodological choice enhances the predictor's ability to discern subtle variations in substrate preferences among different phagolysosomal proteases. The inclusion of diverse structural and physicochemical features such as hydropathy, charge, secondary structure, and solvent accessibility further enriches the predictive model, contributing to a comprehensive assessment of potential cleavage sites.

PredLyP stands out from other available tools by not only predicting the cutting sites of protein fragments but also providing the resulting substrate fragments. Moreover, it implements a sequential cutter functionality that allows proteases to cut sequentially, mimicking biological processes where later proteases act on fragments produced by earlier ones. Future versions of PredLyP could integrate quantitative data on protease frequencies across cell types and maturation stages to further refine predictions, mirroring biological digestion more accurately. In contrast to existing tools, PredLyP outputs both complete and partial protein fragments alongside the positions of cutting sites (P1 locations). These fragments are valuable for antibody development, the study of immune responses, particularly involving HLA-II peptide ligands in dendritic cells (e.g., vaccination and vaccine development), and in pathological conditions, such as autoimmunity [50], [51]. Additionally, they are suited for comparison with mass spectrometry data to validate substrate presence.

Although the addition of physical and structural features did not universally improve overall prediction quality (e.g., negligible F1 gain for CTSK), they provided measurable benefits for certain proteases, such as CTSV and CTSS, where sensitivity was improved. These heterogeneous effects suggest that such features may capture protease-specific preferences not evident across all enzymes. We therefore retained these features in PredLyP to ensure broad applicability and to provide a flexible framework for future improvements as larger training datasets become available.

Furthermore, PredLyP specificity was modestly lower than that of other tools (∼9 % reduction), this trade-off reflects its design focus on sensitivity. In practical applications such as immune-peptidomics, antibody development, or peptide-based vaccine design, capturing the full range of true cleavage events is often more critical than maximizing specificity. Missing potential cleavage sites (false negatives) could overlook biologically relevant peptides, whereas a modest number of additional false positives can be filtered downstream using experimental data. Thus, PredLyP’s sensitivity-oriented balance is advantageous for exploration and translational proteomics research.

A limitation of our current predictor is that it assumes independence among input features, which might not fully reflect reality [52], [53]. Incorporating features that inherently capture these dependencies could enhance predictive performance, as demonstrated by the effectiveness of dipeptide features in classification tasks [54]. Integrating such features into PredLyP would provide a more realistic representation of the data, thereby potentially further improving its predictive accuracy and robustness. Alternatively, advances in deep learning, such as Transformers, have shown promising applications in predicting protein features and interactions [55], [56]. But note that substrate data for most phagolysosomal proteases is limited, hampering these more complex strategies.

Another limitation might be that we currently use SABLE v4 to derive the secondary structure and solvent accessibility features, as newer tools like SPIDER3 [57] or SPOT-1D [58] have shown improved accuracy for these features [59]. Also, for determining feature importance, we have used the Random Forest model, where alternative approaches, such as iterative adjustments of feature weights [60] could be explored.

Additionally, legumain is reported to exhibit pH-dependent specificity, cleaving after both D and N, with cleavage after D favored under neutral conditions [61]. Since phagolysosomes are acidic compartments, this environmental factor could influence cleavage specificity *in vivo*. While the current version of PredLyP does not model pH-dependent effects, we recognize this as a relevant future refinement to increase the biological realism of predictions.

In conclusion, PredLyP represents a unique novel tool for predicting phagolysosomal protease-cutting sites and the resulting protein fragments. Using an optimized selection of regex and PSSMs tailored to these proteases, along with a sequential cutter functionality, PredLyP provides detailed outputs of substrate fragments. This comprehensive toolkit offers valuable support for researchers in proteomics and immunology.

## Author contribution

IK, PD, CT, MJTR and JJMvD designed the study. MW developed the backend of the tool. MW and IK generated the figures, performed the analysis and wrote the first manuscript draft. All authors contributed to the manuscript revision and approved the submitted version.

## Funding

The presented work was funded by the 10.13039/501100000781European Research Council under the European Union’s Horizon 2020 Research and Innovation Programme with an ERC Advanced Grant to JJMvD (ERC-2015-AdG 695655, TiMaScan). CT is the recipient of an Andrés Laguna fellowship (Junta de Castilla y León, co-financed by the Fondo Social Europeo Plus, FSE+; ORDEN EDU/300/2025). PD is supported by a “Ramón y Cajal” fellowship (RYC2022–035568-I; funded by the Spanish Ministry of Science, Innovation and Universities).

## CRediT authorship contribution statement

**Indu Khatri:** Writing – review & editing, Writing – original draft, Visualization, Validation, Supervision, Resources, Project administration, Methodology, Data curation, Conceptualization. **Anniek L. de Jager:** Writing – review & editing, Data curation. **Jacques J.M. van Dongen:** Writing – review & editing, Supervision, Funding acquisition, Conceptualization. **Marcel J.T. Reinders:** Writing – review & editing, Supervision, Conceptualization. **Diez Paula:** Writing – original draft, Supervision, Conceptualization. **Mattijn Wagt:** Writing – review & editing, Software, Formal analysis, Data curation. **Cristina Teodosio:** Writing – review & editing, Writing – original draft, Supervision, Project administration, Methodology, Conceptualization.

## Footnotes

Appendix A Supplementary data associated with this article can be found in the online version at doi:10.1016/j.csbj.2025.10.035.
