# Supplementary material for: PredLyP: A computational tool for predicting tissue-specific (phago-)lysosomal post-digestion peptides
Source: Comput Struct Biotechnol J. 2025 Oct 22;27:4645–56. doi: 10.1016/j.csbj.2025.10.035 (PMC12613027; doi:10.1016/j.csbj.2025.10.035)
Supplement: Supplementary file 1 — Supplementary material [file mmc1.docx]

**PredLyP: A computational tool for predicting tissue-specific**

**(phago-) lysosomal post-digestion peptides**

Mattijn Wagt^1+^, Cristina Teodosio^1,2+^, Anniek L. de Jager^1+^, Jacques J.M. van Dongen^1,2^, Marcel J.T. Reinders^3,4^, Paula Díez^1,5^*, Indu Khatri^1,4^*

+ These authors contributed equally to this work and must be considered as first authors

* Both authors contributed equally to this work and must be considered as senior authors

**Affiliations:**

^1^ Department of Immunology, Leiden University Medical Center, Leiden, The Netherlands.

^2^ Translational and Clinical Research Program, Cancer Research Center (IBMCC, CSIC – University of Salamanca); Cytometry Service, NUCLEUS; Department of Medicine, University of Salamanca (Universidad de Salamanca), Salamanca, Spain; Institute of Biomedical Research of Salamanca (IBSAL), Salamanca, Spain; Biomedical Research Networking Centre Consortium of Oncology (CIBERONC), Instituto de Salud Carlos III, Madrid, Spain.

^3^ Delft Bioinformatics Lab, Delft Technical University, Delft, The Netherlands

^4^ Leiden Computational Biology Center, Leiden University Medical Center, Leiden, The Netherlands.

^5^ Department of Functional Biology (Immunology area), Faculty of Medicine and Health Sciences, University of Oviedo, Oviedo, Spain.

*** Corresponding authors**

J.J.M. van Dongen, MD, PhD: J.J.M.vanDongen@ESLHO.org

Paula Díez, PhD: [diezpaula@uniovi.es](mailto:diezpaula@uniovi.es)

Indu Khatri, PhD: [indu2287@gmail.com](mailto:indu2287@gmail.com)

**Running Title:** PredLyP tool to predict tissue-specific peptides

**Supplementary Table S1: Overview of the (phago)lysosomal proteases present in the available tools for prediction of post-digestion fragments.** (Phago)lysosomal protease availability is marked as blue.

| Proteases | | PeptideCutter, 2002 | SitePrediction, 2009 | PROSPER, 2012 | PROSPERous, 2018 | iProt-Sub, 2019 | ProCleave, 2020 |
| --- | --- | --- | --- | --- | --- | --- | --- |
| SPPL2A | Signal peptide peptidase-like 2A |  |  |  |  |  |  |
| SPPL2B | Signal peptide peptidase-like 2B |  |  |  |  |  |  |
| CTSD | Cathepsin D |  |  |  |  |  |  |
| CTSV | Cathepsin V |  |  |  |  |  |  |
| CTSL | Cathepsin L |  |  |  |  |  |  |
| CTSK | Cathepsin K |  |  |  |  |  |  |
| LGMN | Legumain |  |  |  |  |  |  |
| CTSO | Cathepsin O |  |  |  |  |  |  |
| CTSS | Cathepsin S |  |  |  |  |  |  |
| CTSF | Cathepsin F |  |  |  |  |  |  |
| CTSZ | Cathepsin Z |  |  |  |  |  |  |
| CTSA | Cathepsin A |  |  |  |  |  |  |
| DPP7 | Dipeptidyl-peptidase 2 |  |  |  |  |  |  |
| BACE1 | Beta-secretase 1 |  |  |  |  |  |  |
| CTSC | Cathepsin C |  |  |  |  |  |  |
| CTSH | Cathepsin H |  |  |  |  |  |  |
| CTSB | Cathepsin B |  |  |  |  |  |  |
| CPQ | Carboxypeptidase Q |  |  |  |  |  |  |
| PRCP | Lysosomal Pro-X carboxypeptidase |  |  |  |  |  |  |
| TPP1 | Tripeptidyl-peptidase 1 |  |  |  |  |  |  |


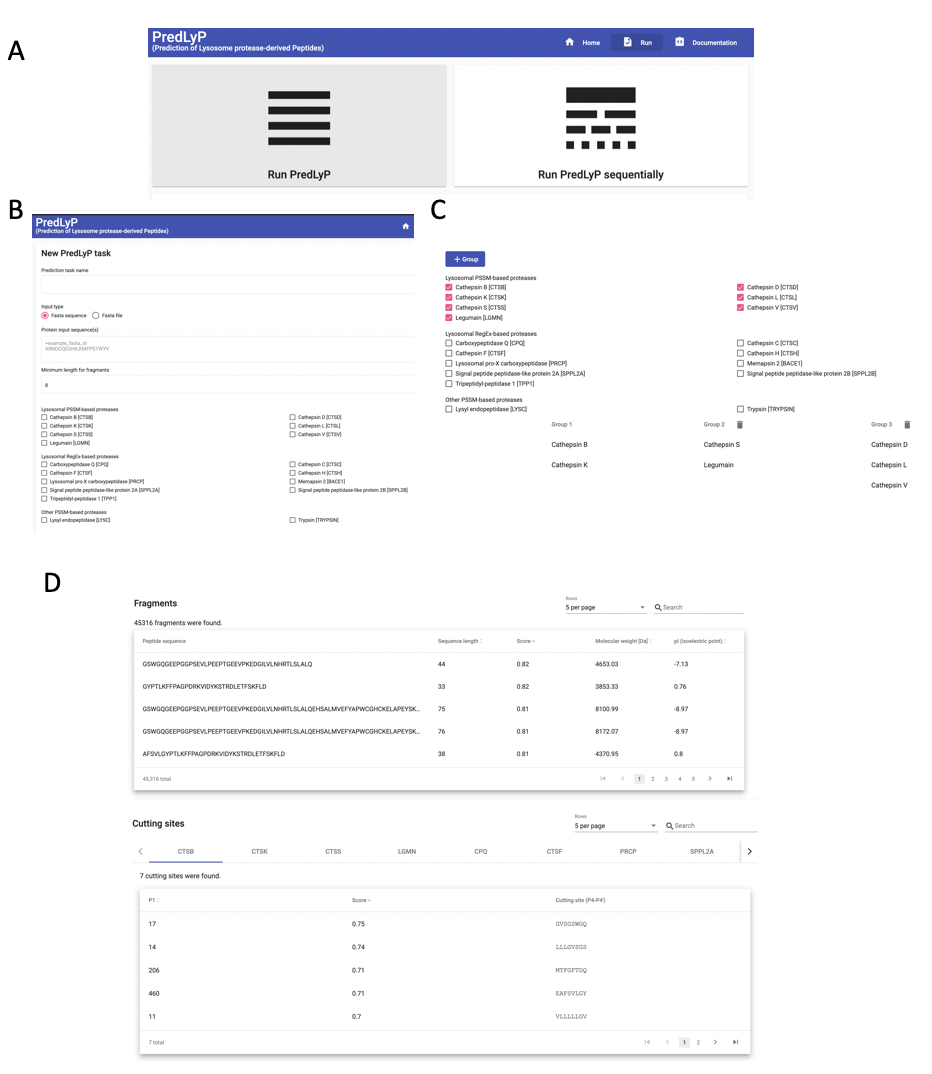


**Supplementary Figure S1**: Interface and workflow of the PredLyP tool for predicting (phago)lysosomal protease-derived peptides. **A)** The main PredLyP interface, showing the two available services: **PredLyP** (single-step prediction mode) and **PredLyP Sequentially** (multi-step sequential prediction mode). The single-step mode predicts fragments from all selected proteases simultaneously, while the sequential mode processes proteases in a stepwise manner based on a user-defined order. **B)** Input parameters for the PredLyP tool, where users configure their prediction task. Parameters include selecting proteases from the list of (phago)lysosomal proteases grouped by PSSM or regex based patterns, and defining the minimum length for the generated fragments. **C)** Input for the **PredLyP Sequentially** service, where users can customize the order of groups, such that proteases in the first group act first, followed by those in subsequent groups on the fragments generated in the earlier steps. **D)** The result page displays the prediction outcomes. The **Fragments table** shows all generated peptide fragments, including details such as sequence length, score, molecular weight, and predicted isoelectric point. This allows users to analyze fragment properties for downstream applications. The **Cutting Sites table** provides an overview of all predicted cutting sites, organized by protease. For each protease, the cutting position (P1) and the surrounding sequence (P4–P4') are displayed alongside a confidence score. This detailed output enables users to examine protease-specific cleavage patterns and the overall digestion profile of the substrate.


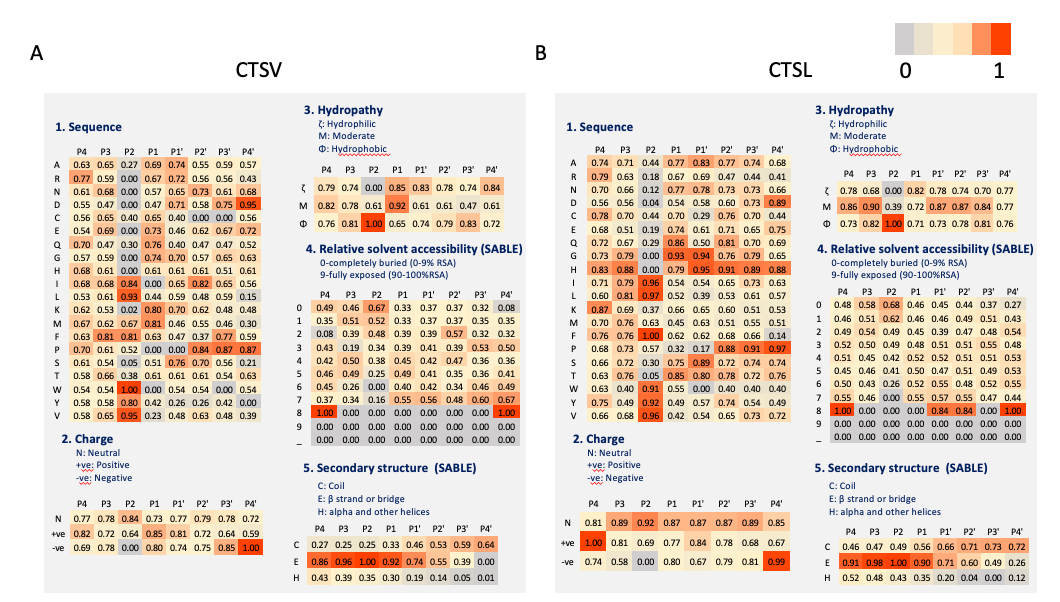


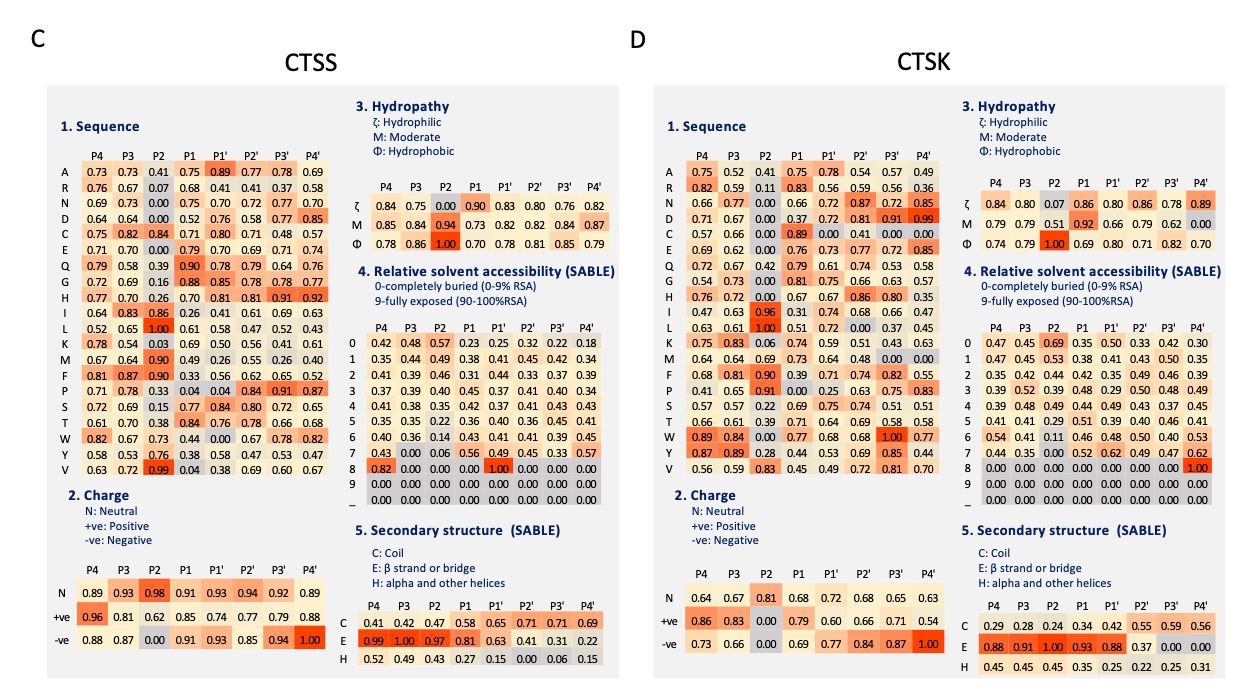


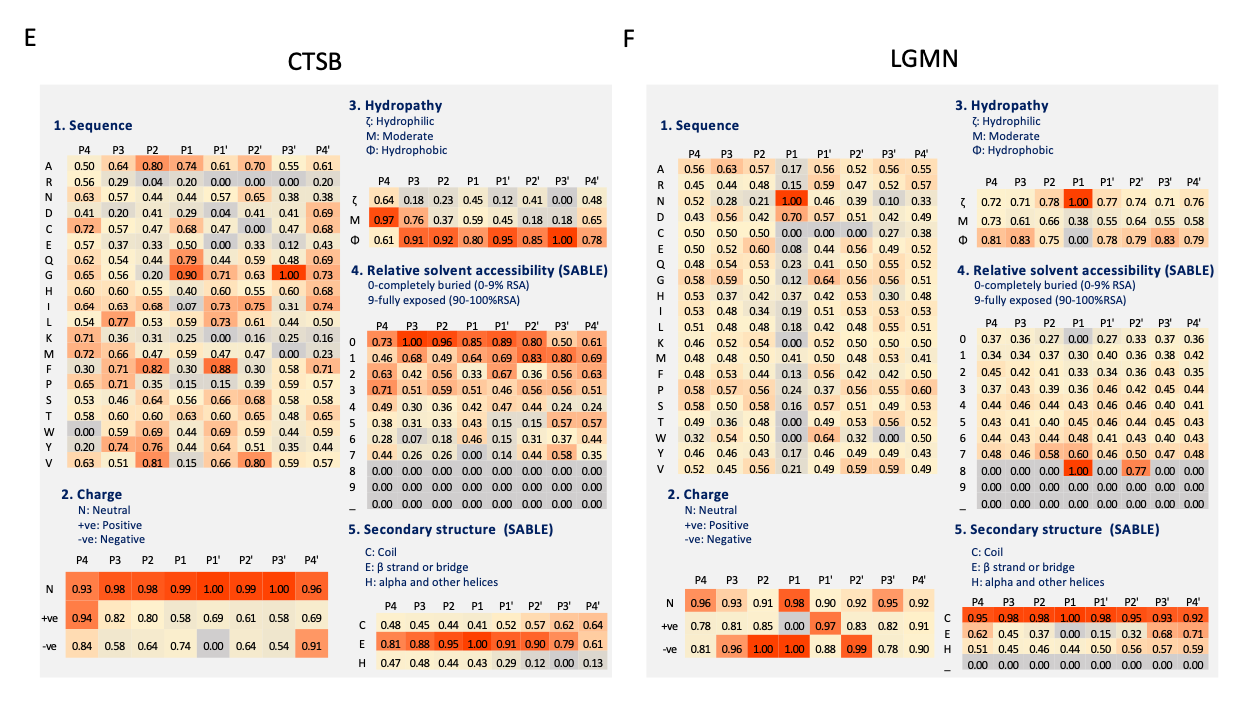


**Supplementary Figure S2**: PSSMs of all the features for the following proteases **A)** CTSV **B)** CTSL **C)** CTSS **D)** CTSK **E)** CTSB and **F)** LGMN.

**
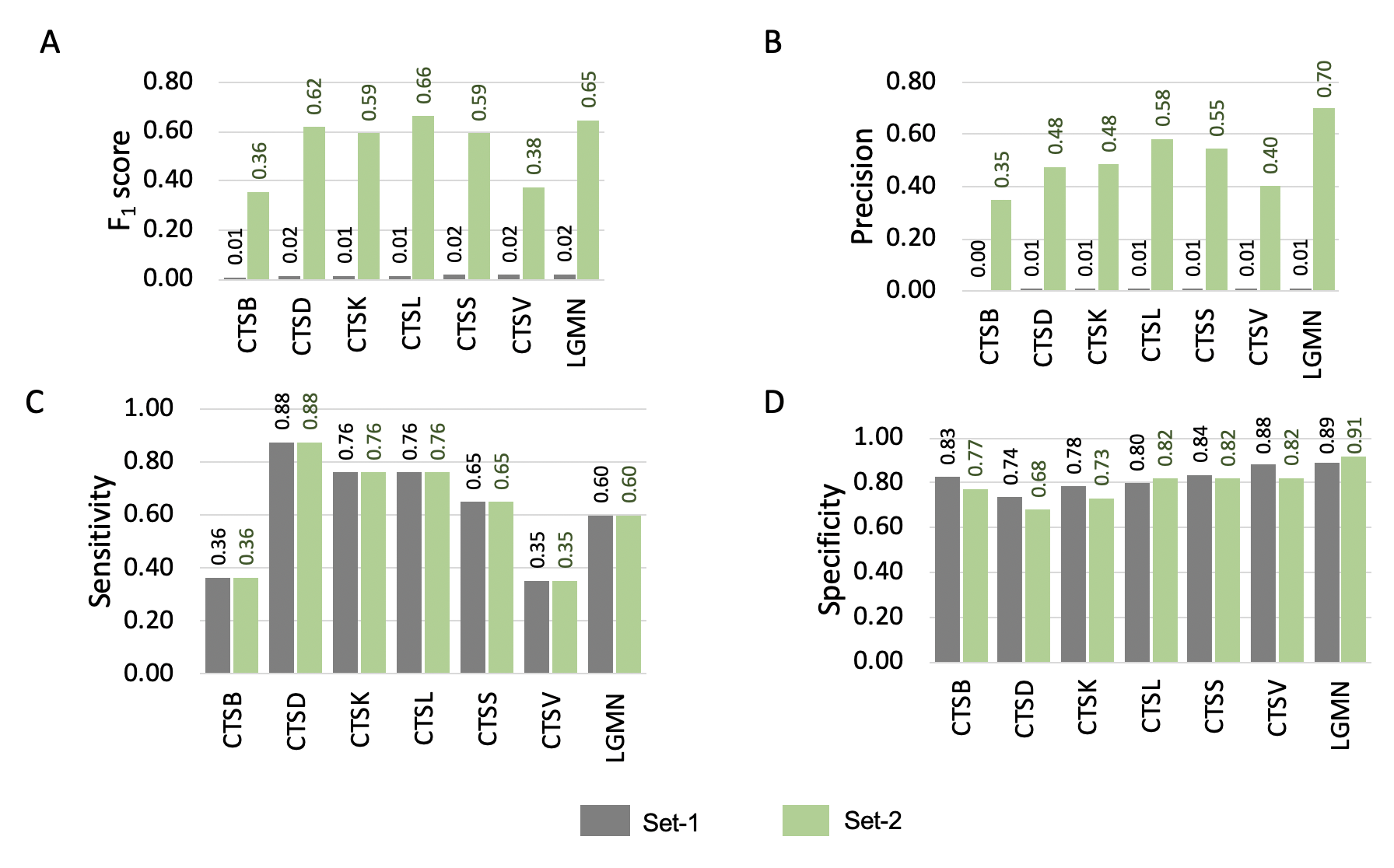
**

**Supplementary Figure S3:** **A)** **F1 score, B) precision, C) sensitivity, and D) specificity comparisons on PredLyP when assessing original (Set-1) and an undersampled (Set-2) dataset.**

**
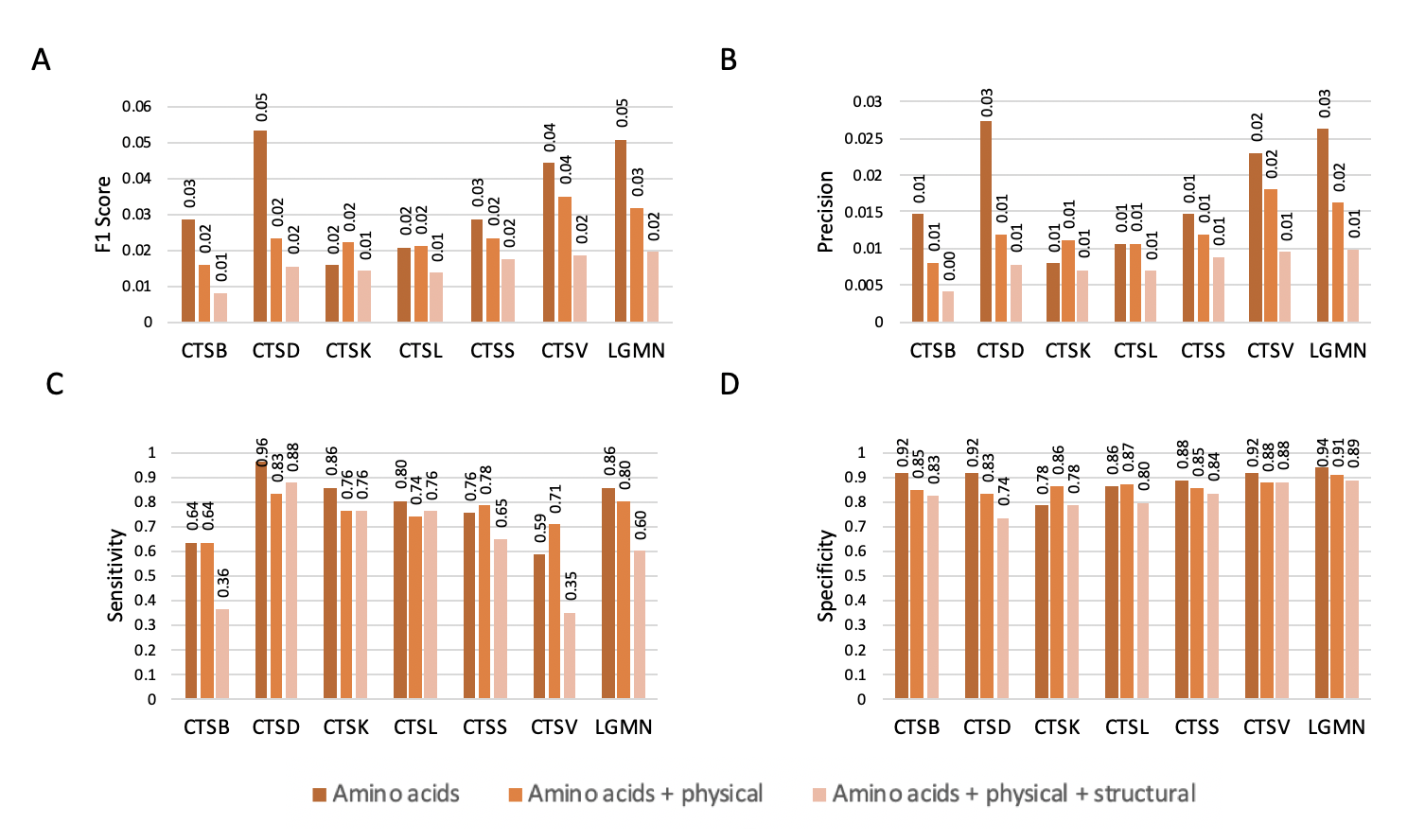
**

**Supplementary Figure S4:** **F1 score, precision, sensitivity, and specificity comparisons on PredLyP using different input features.** Physical refers to charge and hydropathy, and structural refers to secondary structures and solvent accessibility. Set-1 (original) dataset was used for these assessments.

**
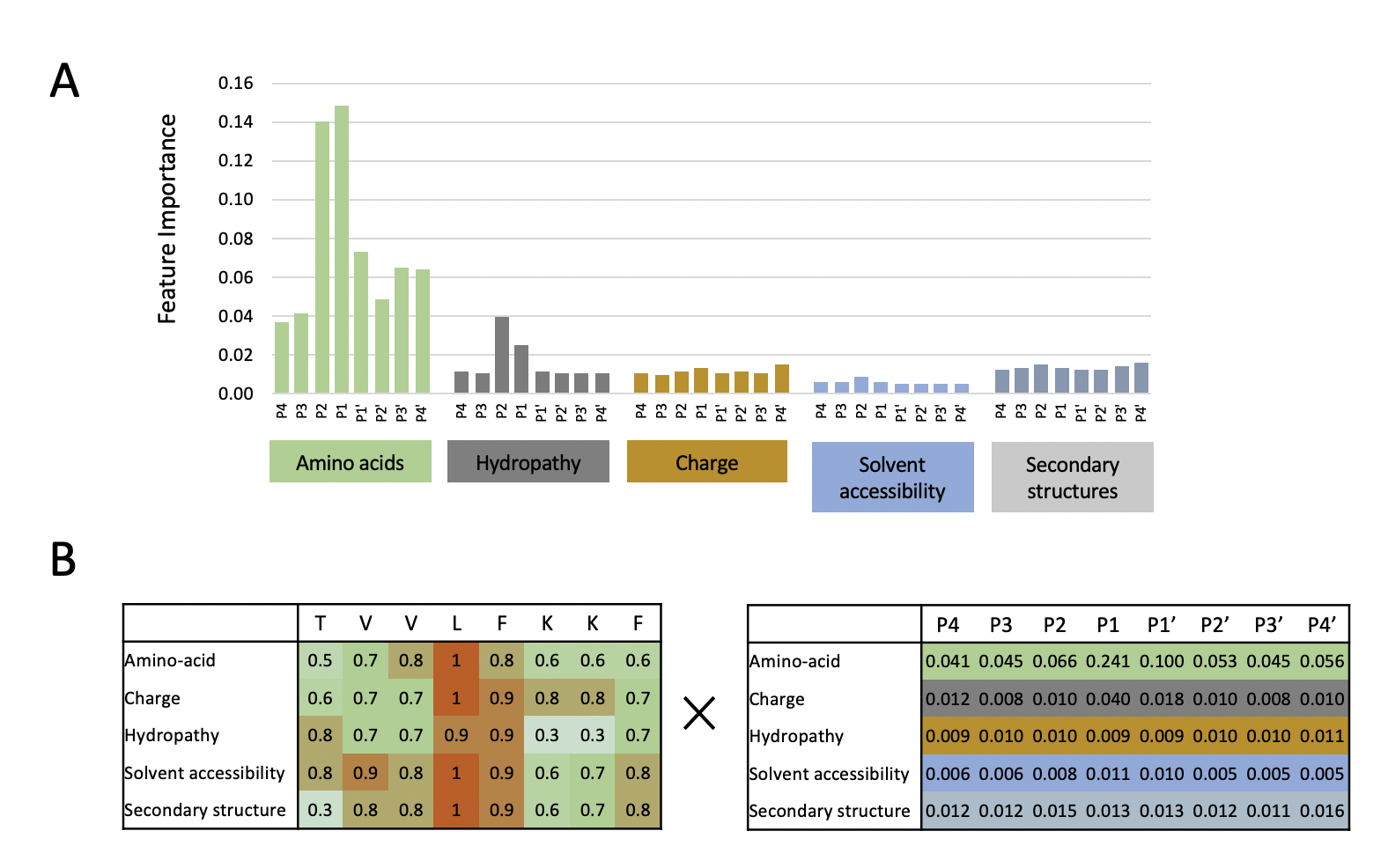
**

**Supplementary Figure S5:** **Using random forest to calculate feature importance and the scores for weighing the features. A)** **Averaged feature importance for each position from P4 to P4’ for each of the five features.** P4 to P4’ are the window/cutting site positions. The features importance for all features sum to 1. Error bars refer to standard error. **B)** **Multiplication of the window scores,** for all five features, against the obtained feature importances.

**
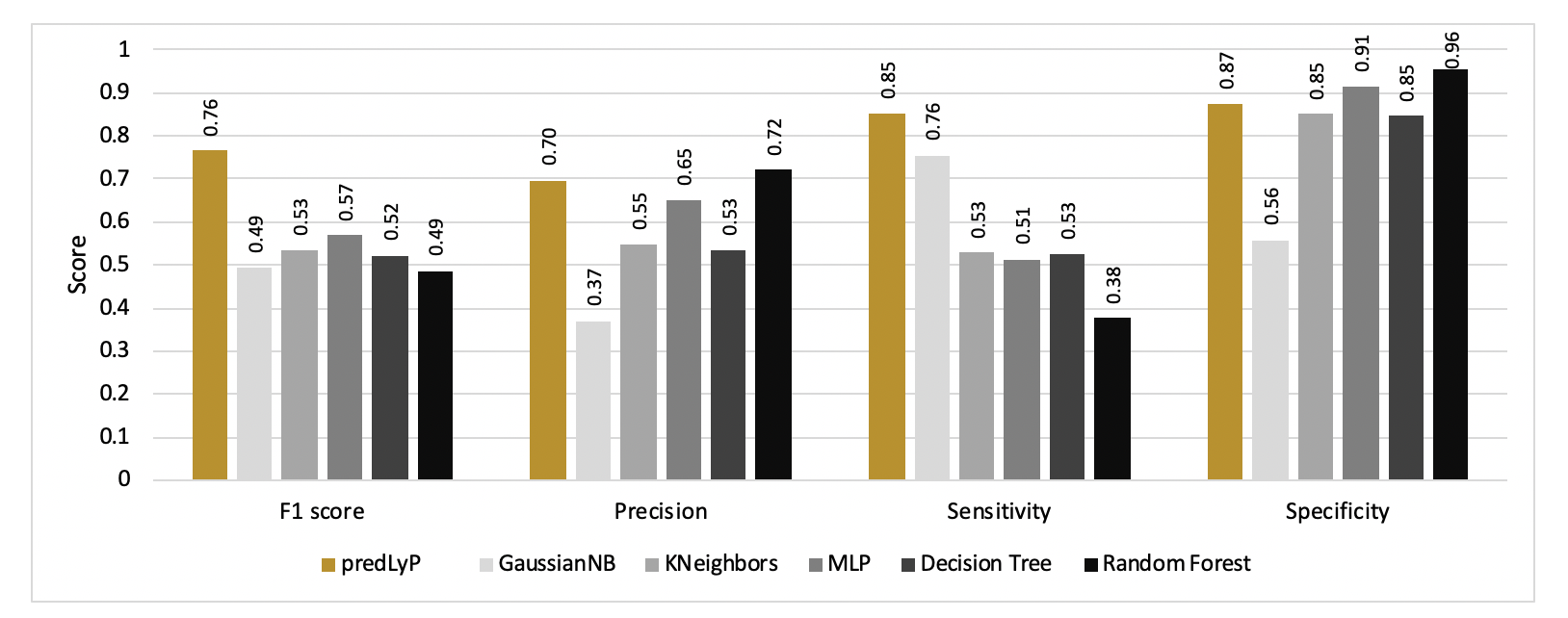
**

**Supplementary Figure S6**: **Comparison between PredLyP (Yellow) and different machine learning classifiers (different shades of grey).** Comparison is made using Set-2 and only the amino acid features were used. The average values of all the proteases are shown here.

**
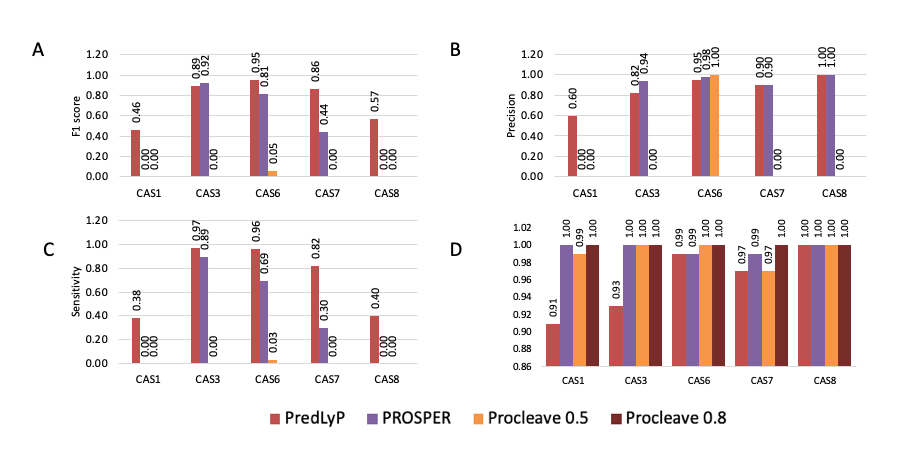
**

**Supplementary Figure S7**: **F1 score, precision, sensitivity, and specificity comparisons between PredLyP v2, PROSPER and ProCleave using Caspase 1, 3, 6, 7 and 8**. Comparisons were made using Set-2 i.e. downsampled data with better signal-to-noise ratio. For ProCleave score thresholds of 0.5 and 0.8 were used as suggested by ProCleave. F1 score, Precision and Sensitivity were 0 for all Procleave 0.5 and 0.8.
